# A low-damage electric harvesting system for lodging-prone *Cerasus humilis*: design, optimization and field validation

**DOI:** 10.3389/fpls.2026.1904743

**Published:** 2026-08-11

**Authors:** Yongqiang He, Yanqing Zhang, Liyang Zhao, Jianguo Yang, Dongjin Yang, Junlin He

**Affiliations:** 1 College of Agricultural Engineering, Shanxi Agricultural University, Taigu, China; 2 Shanxi Key Laboratory of Key Technologies and Equipment for Agricultural Machinery, Taigu, China; 3 School of Mechanical Engineering, Xijing University, Xi’an, China

**Keywords:** branch lifting, *Cerasus humilis*, comb stripping, electric harvester, field validation, low-damage harvesting, response surface methodology

## Abstract

Mechanized harvesting remains a major bottleneck for sustainable *Cerasus humilis* production because lodging-prone branches and fragile fruit make conventional small-fruit harvesters difficult to adapt. To address this crop-specific constraint, we developed a low-damage self-propelled electric harvesting system that integrates branch lifting, comb-type stripping, screw conveying, self-propelled travel and electrohydraulic height adjustment. The key working modules were analyzed, and a four-factor, three-level Box-Behnken field experiment was used to optimize travel speed and the rotational speeds of the branch-lifter, stripping and conveying motors. Travel speed had the strongest effect on harvesting rate, whereas stripping speed had the strongest effect on fruit damage. The optimized operating settings were a travel speed of 0.2 m/s, a branch-lifter speed of 104.6 r/min, a stripping motor speed of 22 r/min and a conveying motor speed of 137.4 r/min. Five validation tests under these settings achieved a mean harvesting rate of 82.12% and a mean damage rate of 2.79%, compared with predicted values of 81.26% and 2.80%, respectively. These results indicate that coordinated electric control of branch lifting, comb-type stripping and fruit conveying enables sustainable low-damage field harvesting of *C. humilis* under the tested cultivar and site conditions.

## 1 Introduction

*Cerasus humilis*, also known as Chinese dwarf cherry or calcium fruit, is a perennial deciduous shrub native to northern China. The crop has gained agronomic value because its fruit combines distinctive acidity, high calcium accumulation, diverse volatile compounds and substantial quality variation (Guo et al., 2025; Zhao et al., 2022; Zhang et al., 2023). Studies of organic-acid metabolism, sugar-calcium accumulation, methyl jasmonate responses, and root development further support its potential for breeding, cultivation, and orchard establishment (Guo et al., 2022; Zhang et al., 2024, 2025; Yin et al., 2024). Planting has expanded in North and Northwest China, but the fruit ripens within a short harvest window. Delayed harvest accelerates softening, quality deterioration and postharvest defects (Wang et al., 2024a; Wang and Li, 2023).

Harvesting is therefore a critical constraint on sustainable *C. humilis* production. Current practice still relies mainly on manual picking, which is labor-intensive, costly and difficult to scale during the concentrated ripening period. The crop bears dense fruit on flexible branches that lodge close to the ground, slowing fruit removal and increasing the risk of compression and impact injury. Efficient field machinery must therefore solve two linked plant-production problems: raising lodged branches into a stable picking posture and removing fruit without excessive mechanical damage (Hao et al., 2025; Tong et al., 2024; Liu et al., 2021).

Previous *C. humilis* studies provide the mechanical basis for such a system but do not yet provide a complete field solution. Compression-fracture simulations and temperature-dependent tests have supplied parameters for predicting fruit tissue failure (Hao et al., 2025; Tong et al., 2024). Bench-scale combing, free-fall impact, and single-branch combing experiments have helped define comb geometry, contact behavior, and low-loss detachment conditions (Liu et al., 2021; Kang et al., 2023, 2024). These studies show that harvest rate and damage rate depend on coordinated control of branch posture, comb contact, fruit receiving, and conveying, rather than on a single detachment mechanism alone. The above studies were mostly conducted under test conditions, which could not fully reproduce the complex working conditions such as uneven posture of fallen branches in the field, disturbance of the whole machine during movement, and continuous collection and transportation of fruit. Therefore, they were difficult to directly support parameter matching for whole machine field operations.

The remaining gap is field integration. Most available work has focused on laboratory mechanisms, test benches, or isolated components, while few studies have tested a self-propelled machine that can lift lodged branches, detach fruit, collect and convey the crop, and continue operating in orchard rows. Intermittent or walk-behind equipment also limits throughput when the harvest window is short. A dedicated electric harvester is needed to connect low-damage detachment with continuous field operation.

Recent advancements in agricultural engineering have heavily focused on precision agricultural robotics, AI-assisted visual perception, and autonomous end-effectors for fruit harvesting (Wang et al., 2022). In the context of Agriculture 4.0, deep learning models like fine-tuned YOLOv8 are now widely utilized for crop state detection, supporting vision-guided robotic arms and adaptive end-effectors in achieving non-destructive harvesting (Verma et al., 2024; Paul and Machavaram, 2025d; Paul et al., 2026). While these intelligent systems offer non-destructive picking for greenhouse crops and large tree fruits, they often face significant throughput bottlenecks when applied to small, densely clustered, and severely lodged shrub fruits like C. humilis. Therefore, balancing continuous field capacity with low-damage detachment remains a critical research gap.

Research on other berry and woody-fruit crops offers useful principles, but their machines cannot be transferred directly to *C. humilis* because harvesting response is strongly shaped by crop morphology, branch flexibility, and fruit-stem interactions. In goji berry, recent studies have optimized harvester parameters through response surface methods and field tests, clarifying how vibration amplitude, frequency, and machine posture affect fruit detachment and damage (Wang et al., 2024b; Yu et al., 2024; Chen et al., 2024a). Other work on vibratory systems, pneumatic oscillators, and compliant mechanisms has used kinematic analysis and modal analysis to improve excitation transmission and reduce mechanical injury (Chen et al., 2024b; Zhao et al., 2021; Chen et al., 2021a). These findings also indicate that the dynamic coupling among actuator, branch, and fruit is highly crop-specific, so mechanisms developed for goji berry cannot automatically satisfy the structural constraints of *C. humilis* (Chen et al., 2022; Chen et al., 2021b). Studies on comb-brush and canopy-shaking systems in Camellia oleifera, olive, and rape further show that harvesting efficiency depends on the compatibility between the picking unit and canopy or branch architecture (Du et al., 2021, 2022; Wu et al., 2022). Similar conclusions have been reported for blueberry and related fruit crops, where fruit maturity, detachment force, and tissue mechanical properties strongly influence loss rate and damage level (Yan et al., 2025; Niu et al., 2022; Zhan et al., 2022). Therefore, although these studies provide useful methodological references, the harvesting mechanism for *C. humilis* still needs to be designed around its own plant structure and fruit mechanical characteristics (Brondino et al., 2021).

*C. humilis* presents a different design problem because its low canopy, severe lodging, compact fruiting zones, and thin peel make uncontrolled contact especially damaging. A practical harvester must first guide lodged branches upward, then apply controlled combing to detach fruit, and finally convey the fruit away while limiting secondary impact. This requirement motivated an integrated electric system in which travel, branch lifting, stripping, conveying, and height adjustment can be coordinated during field operation.

In this study, we designed and field-tested a low-damage self-propelled electric harvesting system for *C. humilis*. The objectives were to develop the integrated machine architecture, analyze the working principles of the branch-lifting, comb-type stripping, conveying, chassis, and control modules, and optimize operating parameters for harvesting rate and fruit damage rate. This study proposes a sustainable low-damage harvesting approach for lodging-prone fruit shrubs.

## 2 Materials and methods

### 2.1 Structure and working principle of the whole machine

**Figure 1:**
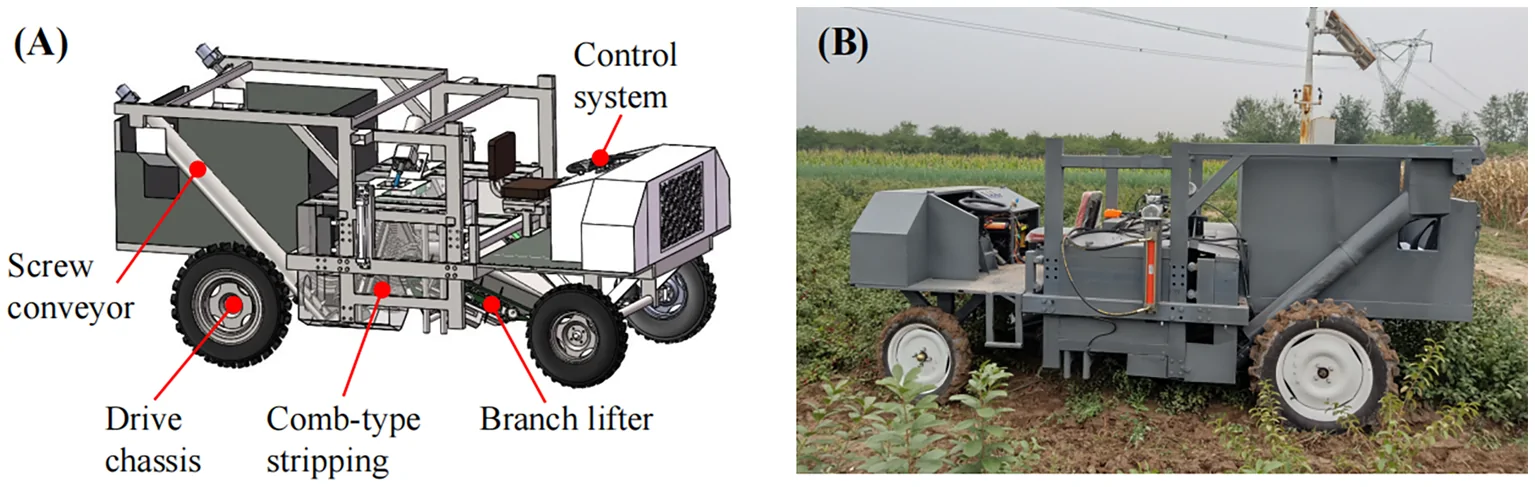
Self-propelled electric harvester for *C. humilis.* **(A)** The 3D structural diagram. **(B)** The prototype.

The harvester was designed as an integrated electric field platform for low-canopy, lodging-prone shrub fruit. It consists of five functional modules: branch lifter, comb-type stripping device, screw conveyor, walking chassis and centralized control system (**Figure 1**). The design sequence followed the harvest process, from branch guidance to fruit detachment, fruit transfer, field travel and working-height regulation.

The prototype consists of five functional modules: branch lifter, comb-type stripping device, screw conveyor, drive chassis and control system. **Figure 1A** shows the overall structure, and **Figure 1B** shows the manufactured prototype. During operation, the branch lifter first raises and aligns fruit-bearing branches before they enter the comb-type stripping zone. Rotating comb teeth detach the fruit, and the screw conveyor then transfers the harvested fruit to the collection carriage. Before entering a row, the electrohydraulic lifting mechanism sets the working height of the lifter, stripping device and conveyor. The three working motors are regulated by frequency converters, while forward speed is adjusted through the electronic throttle. This arrangement allows branch posture, detachment intensity, and fruit transport to be coordinated during continuous field harvesting.

### 2.2 Design of the branch lifter

**Figure 2:**
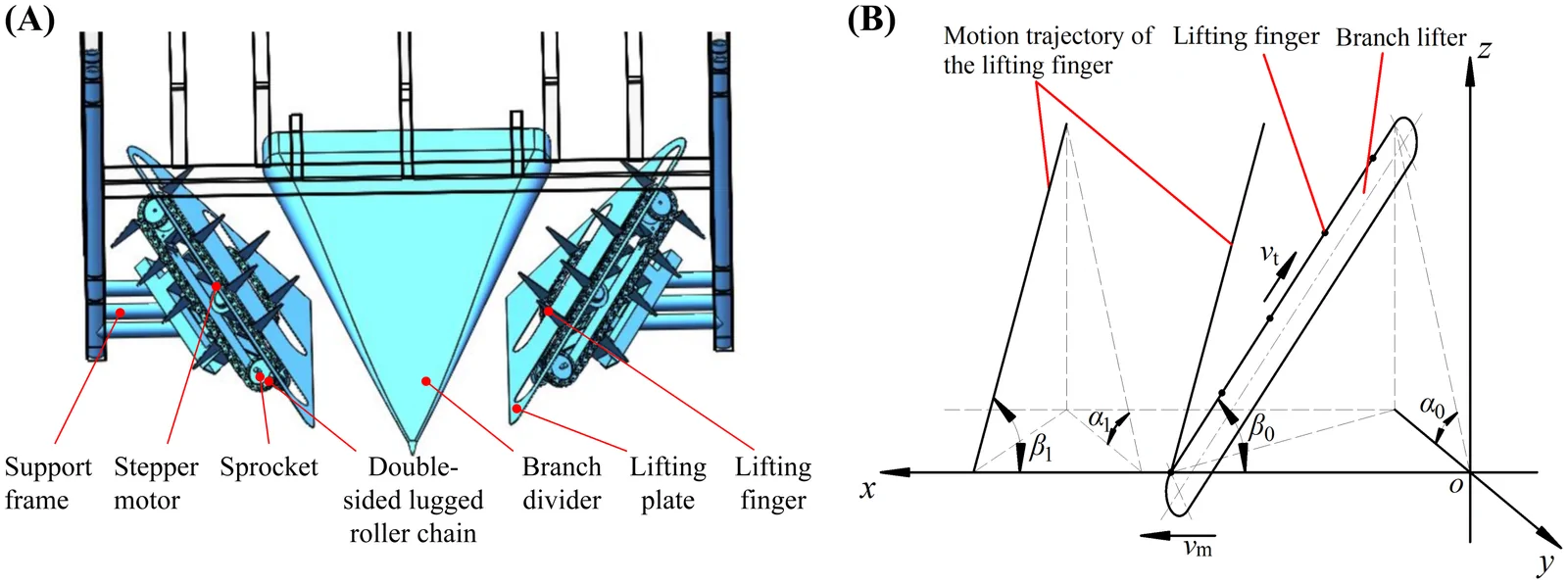
The branch lifter. **(A)** The three-dimensional structure of the branch lifter. **(B)** The movement trajectory of the lifting-finger.

The branch lifter is the front-end guidance unit that converts lodged branches into a harvestable posture. As shown in **Figure 2A**, it consists of a support frame, stepper motor, sprockets, double-sided lugged roller chain, branch dividers, lifting plates and lifting fingers. Its geometry and motion parameters were designed to influence both the probability of fruit entering the stripping zone and the risk of mechanical injury. Because mature *C. humilis* branches are heavy and often lie near the soil surface, the lifting fingers must enter beneath the canopy, raise the branches and hold them upright as the machine advances. Each finger follows the chain around the upper and lower sprockets, and one cycle includes raking, lifting, and idle return. The lifting trajectory results from the combined motion of the finger and the harvester.

As shown in **Figure 2B**, the direction of the lifting-finger trajectory is calculated from the finger linear velocity, the harvester forward speed, the elevation angle and the deflection angle, as shown in Equations 1–3.

(1) $$\alpha_{1}=\alpha_{0}$$

(2) $$\beta_{1}=\arctan\frac{\sin\beta_{0}}{\cos\beta_{0}+\frac{v_{\text{m}}}{v_{\text{t}}}}$$

(3) $$v_{t}=\pi n_{1}(d_{1}+2h)$$

where *n*_1_ denotes the sprocket rotational speed, r/min, *d*_1_ is the sprocket tip diameter, 0.092 m and *h* is the lifting-finger length, m. The displacement of the lifting-finger tip during the lifting stage is then described by Equation 4:

(4) $$\left\{ \begin{array}{l} X=(v_{\text{t}}\cos\beta_{0}+v_{\text{m}})t\\ Y=v_{\text{t}}\sin\beta_{0}\cos\alpha_{0}t\\ Z=v_{\text{t}}\sin\alpha_{0}\sin\beta_{0}t \end{array}\right.$$

where *X*, *Y* and *Z* are the time-dependent displacements of the lifting finger tip along the positive *x*, *y* and *z* axes, respectively, and *t* is the duration of the branch-lifting process, s. *v*_m_ is the forward speed of the harvester relative to the branches, m/s; *v*_t_ is the linear velocity of the lifting finger, m/s; *α*_0_ is the elevation angle, (°); *β*_0_ is the deflection angle, (°); *α*_1_ is the angle between the branch-lifting trajectory of the lifting finger and the horizontal plane, (°); *β*_1_ is the angle between the branch lifting trajectory of the lifting finger and the velocity direction of the branches, (°).

### 2.3 Design of the comb-type stripping device

#### 2.3.1 Overall design and working principle

**Figure 3:**
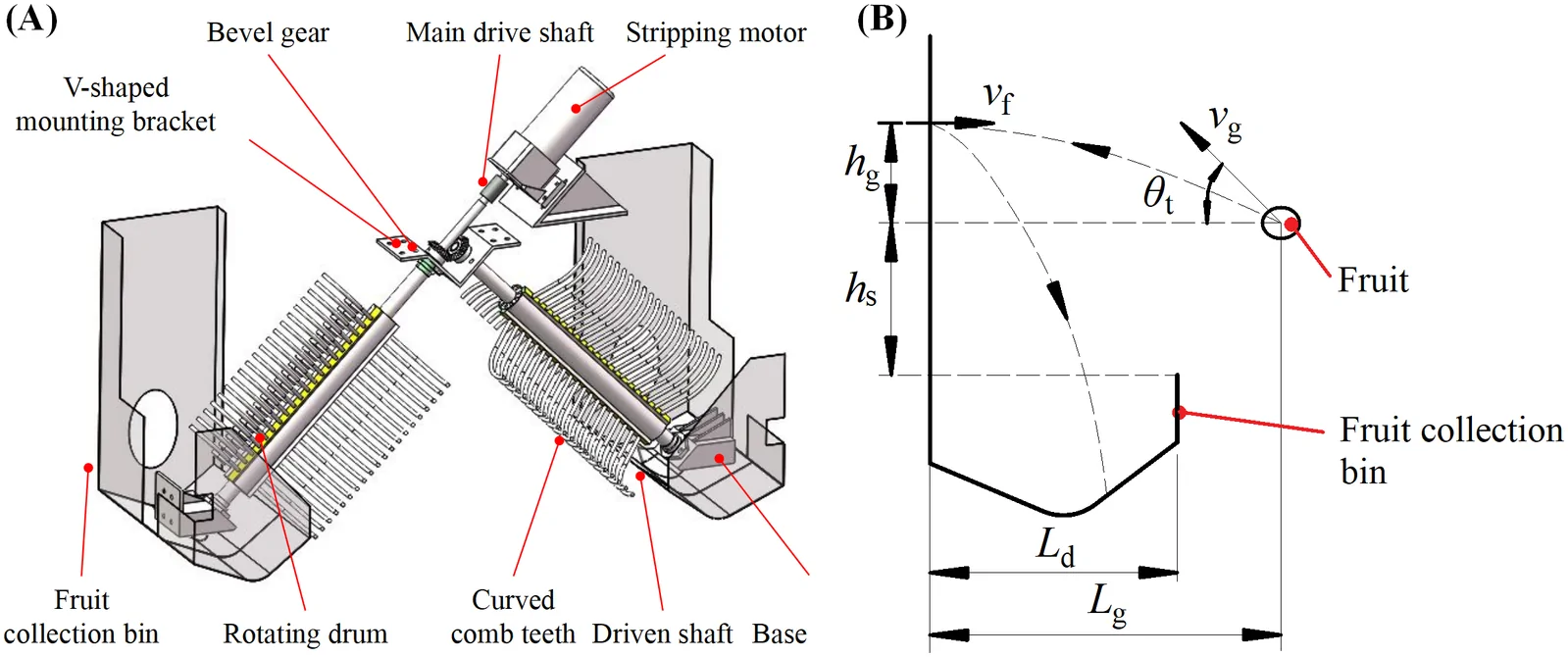
Schematic diagram of the comb-type stripping device. **(A)** The three-dimensional structure of the comb-type stripping device. **(B)** Schematic of motion trajectory during fruit collision with the collection bin.

Compared to vibratory systems which suffer from severe excitation attenuation in flexible lodged branches, or robotic end-effectors and soft grippers that face severe throughput limitations in dense fruit clusters, the comb-type mechanism was selected as it provides a continuous, high-efficiency harvesting mode while maintaining detachment forces within an acceptable threshold for thin-peeled fruits. The comb-type stripping device performs the fruit-detachment step after branch posture has been adjusted. Rotating curved teeth sweep the fruiting branches and separate fruit from the pedicels. As shown in **Figure 3A**, the device includes a stripping motor, main drive shaft, driven shaft, bevel gears, V-shaped mounting bracket, rotating drum, curved comb teeth, base and fruit collection bin. The main and driven shafts are connected by bevel gears at a right angle. Three rows of curved comb teeth are arranged evenly around the rotating drum, with 0.02 m spacing between adjacent teeth. During harvesting, branches pass through the tooth gaps while fruit is intercepted by the comb teeth. Detachment occurs when the combing force exceeds the fruit-pedicel binding force, after which the fruit is guided into the receiving bin.

As shown in **Figure 3B**, After release, the fruit may strike the inner wall of the collection bin, rebound and then fall into the bin under gravity. This motion path was modeled to reduce fruit scattering and secondary impact during collection. As shown in Equation 5, the initial velocity *v*_g_ of the fruit is approximated by the linear velocity at the contact point on the curved comb teeth:

(5) $$v_{\text{g}}=\omega_{\text{t}}r=2\pi n_{\text{s}}r$$

where *v*_g_ is the initial velocity of the fruit after release, m/s; *ω*_t_ is the angular velocity of the comb shaft, rad/s; *r* is the radial distance between the fruit contact point and the comb axis, m; and *n*_s_ is the rotational speed of the comb shaft, r/min.

Because fruit can contact the bin wall at different angles, the model assumed perpendicular impact with the inner wall. The flight from detachment to wall contact was treated as reverse horizontal projectile motion. The flight time *t*_1_, rising height *h*_s_, and impact velocity *v*_p_ are given by Equations 6–8.

(6) $$t_{1}=\frac{L_{\text{g}}}{v_{\text{g}}\cos\theta_{\text{t}}}$$

(7) $$h_{\text{g}}=\frac{1}{2}g{t_{1}}^{2}$$

(8) $$v_{\text{p}}=v_{\text{g}}\cos\theta_{\text{t}}$$

where *t*_1_ is the time from fruit detachment to contact with the inner wall, s; *L*_g_ is the initial distance between the fruit and the inner wall, m; *θ*_t_ is the angle between *v*_g_ and the horizontal plane, 45°; *h*_g_ is the rising height, m; *g* is gravitational acceleration, 9.8 m/s^2^; *v*_p_ is the impact velocity, m/s.

Energy loss during wall contact was represented by the deformation-related velocity loss coefficient *k*_s_. With *k*_s_ set to 0.3 (He et al., 2019), the rebound velocity was calculated by Equation 9:

(9) $$v_{\text{f}}=k_{\text{s}}v_{\text{p}}$$

where *v*_f_ is the initial rebound velocity of the fruit after collision with the inner wall, m/s; *k*_s_ is the coefficient of restitution for the collision between the fruit and the steel plate.

After rebound, *v*_f_ was assumed to act horizontally, and the subsequent fruit path was modeled as horizontal projectile motion. The time *t*_2_ required for the fruit to reach the height of the upper edge of the right inner wall is expressed by Equation 10:

(10) $$\frac{1}{2}g{t_{2}}^{2}=h_{\text{g}}+h_{\text{s}}$$

where *t*_2_ is the time required for the fruit to rebound from the collision point to the horizontal level of the upper edge of the right inner wall, s; *h*_s_ is the vertical height from the fruit release point to that level, m.

For the fruit to enter the collection bin after rebound, the Equation 11 must be satisfied:

(11) $$t_{2}<\frac{L_{\text{d}}}{v_{\text{f}}}$$

From Equations 6–11, Equations 7–12:

(12) $${k_{\text{s}}}^{2}(1+\frac{h_{\text{s}}}{h_{\text{g}}})<\frac{{L_{\text{d}}}^{2}}{{L_{\text{g}}}^{2}}$$

where *L*_d_ is the width of the fruit collection bin, m. Equation 12 indicates that fruit recovery improves when the collection-bin width *L*_d_ increases and the initial distance *L*_g_ from the wall decreases. To increase bin capacity without interfering with the curved teeth, *L*_d_ was set to 0.335 m. Based on the possible branch-entry positions in the combing zone, *L*_g_ ranged from 0.25 to 0.56 m at fruit detachment.

#### 2.3.2 Analysis of forces acting on *C. humilis* fruit during the stripping process

**Figure 4:**
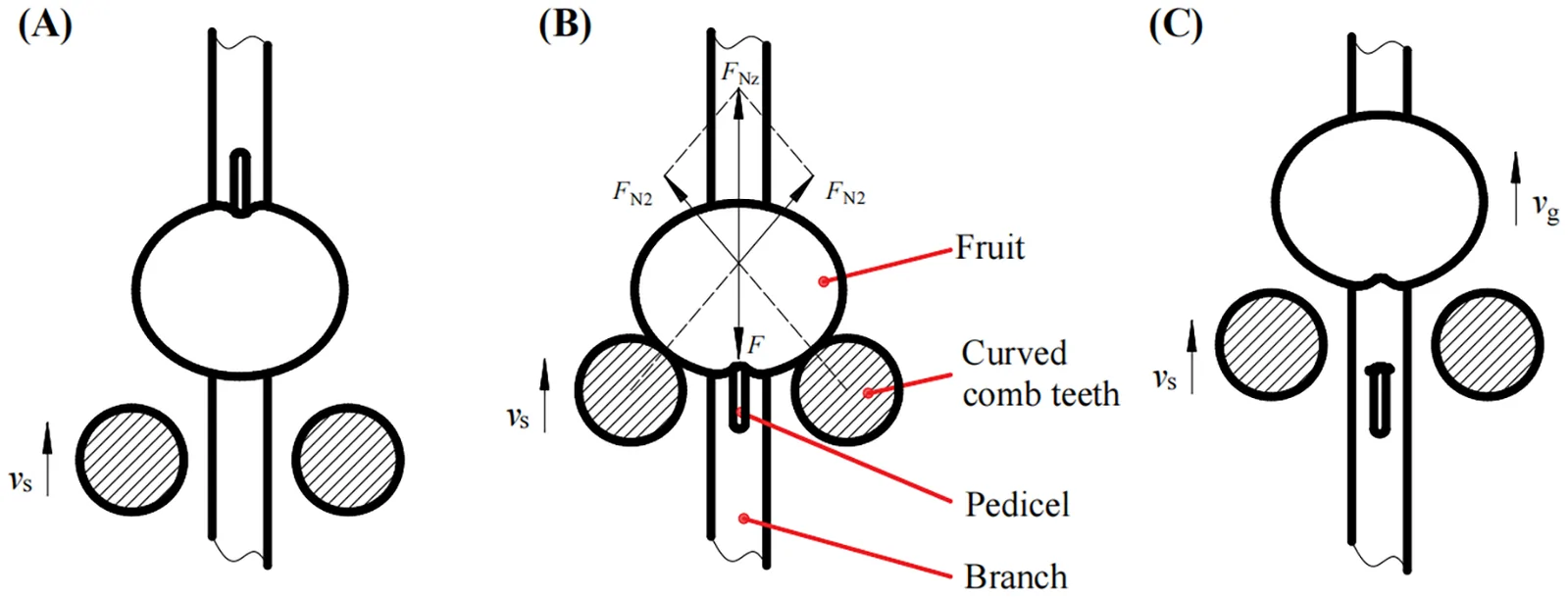
Force analysis of *C. humilis* fruit during the stripping process. **(A)** Before collision. **(B)** During collision. **(C)** After collision.

During the stripping process, the curved teeth push fruit-bearing branches upward. Before impact, each fruit remains connected to the branch by its pedicel and hangs naturally, as illustrated in **Figure 4A**.

When a comb tooth contacts a fruit, it applies an upward impact with velocity ${\text{v}}_{\text{s}}$ and stretches the pedicel (**Figure 4B**). The fruit is released with initial velocity ${\text{v}}_{\text{g}}$ when the force condition in Equation 13 is satisfied (**Figure 4C**).

(13) $$F_{\text{Nz}}>F_{\text{j}}$$

where *F*_Nz_ is the resultant force exerted by the curved comb teeth on the *C. humilis* fruit, N; *F*_j_ is the binding force between the pedicel and fruit, N.

It is important to note that the force analysis presented in **Figure 4** is a simplified boundary model. Under actual field conditions, the detachment mechanism is a dynamic process highly influenced by impact duration, the damping effect of flexible branches, and variations in fruit maturity, all of which alter the ultimate pedicel-binding force.

### 2.4 Design of the screw conveyor

#### 2.4.1 Overall design and working principle

The screw conveyor transfers detached fruit from the receiving bin to the storage compartment. Driven by the conveying motor, the screw shaft moves fruit axially while the helical blade and pipe wall constrain circumferential motion. This compact layout was selected because it provides stable transport, adjustable speed and a short transfer path. The screw conveyor consists of a conveying motor, coupling, rotating shaft, helical blade and conveying pipe.

#### 2.4.2 Force and motion analysis of *C. humilis* fruit during screw conveying

The screw shaft and helical blade form a welded assembly, and motor torque is transmitted through a coupling. During steady operation, the shaft rotates at angular velocity *ω*_s_. Under blade support, gravity, and centrifugal effects, fruit moves along the inner wall of the pipe and advances axially when the axial component of blade force exceeds friction. The resulting path can be regarded as a spatial spiral.

**Figure 5:**
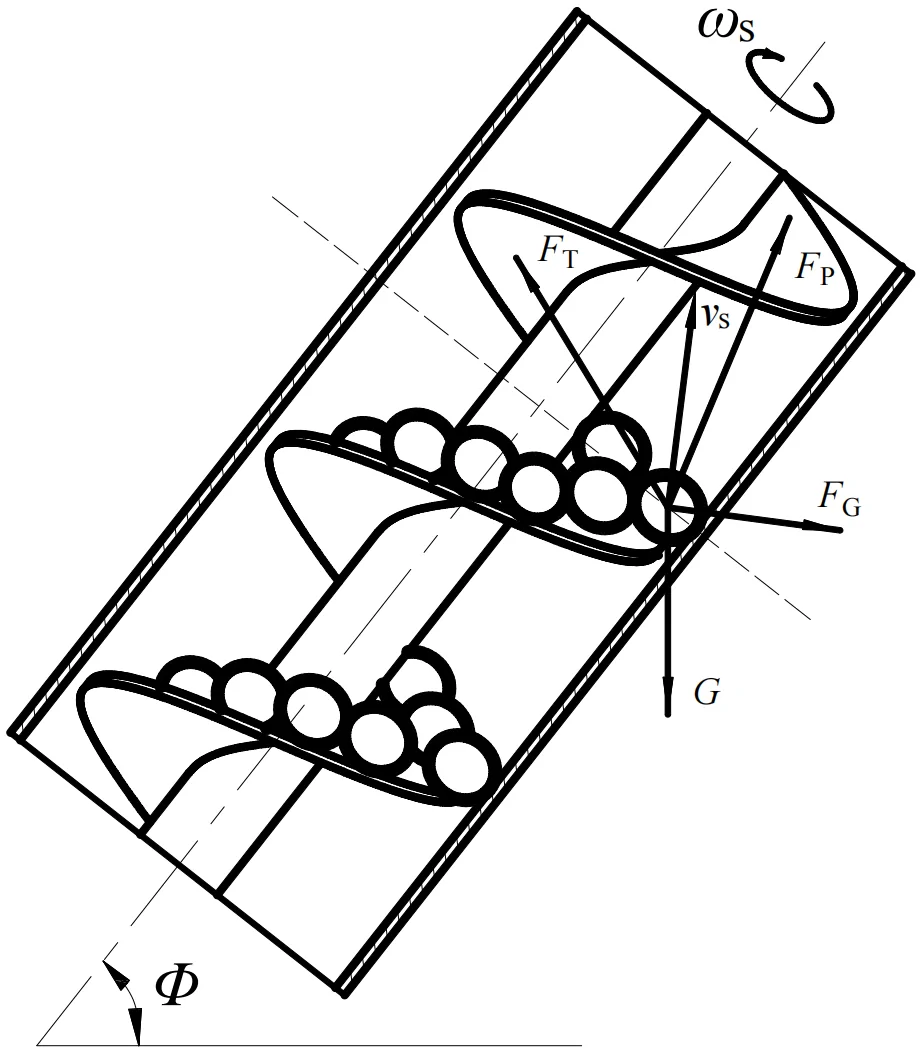
Force analysis of *C. humilis* fruit during screw conveying.

A single *C. humilis* fruit near the inner wall of the conveying pipe was selected as the analysis object. **Figure 5** shows the force distribution and velocity directions during conveying. The fruit is acted on by the helical-blade force *F*_P_, pipe-wall force *F*_T_, interaction force *F*_G_ from adjacent fruit and gravity *G*. F_P_ contains the blade-surface friction *f*_P_ and normal support *F*_NP_, *F*_T_ contains wall friction *f*_T_ and normal support *F*_NT_, and *F*_G_ contains the normal extrusion force *F*_NG_ and friction *f*_G_ between adjacent fruits.

**Figure 6:**
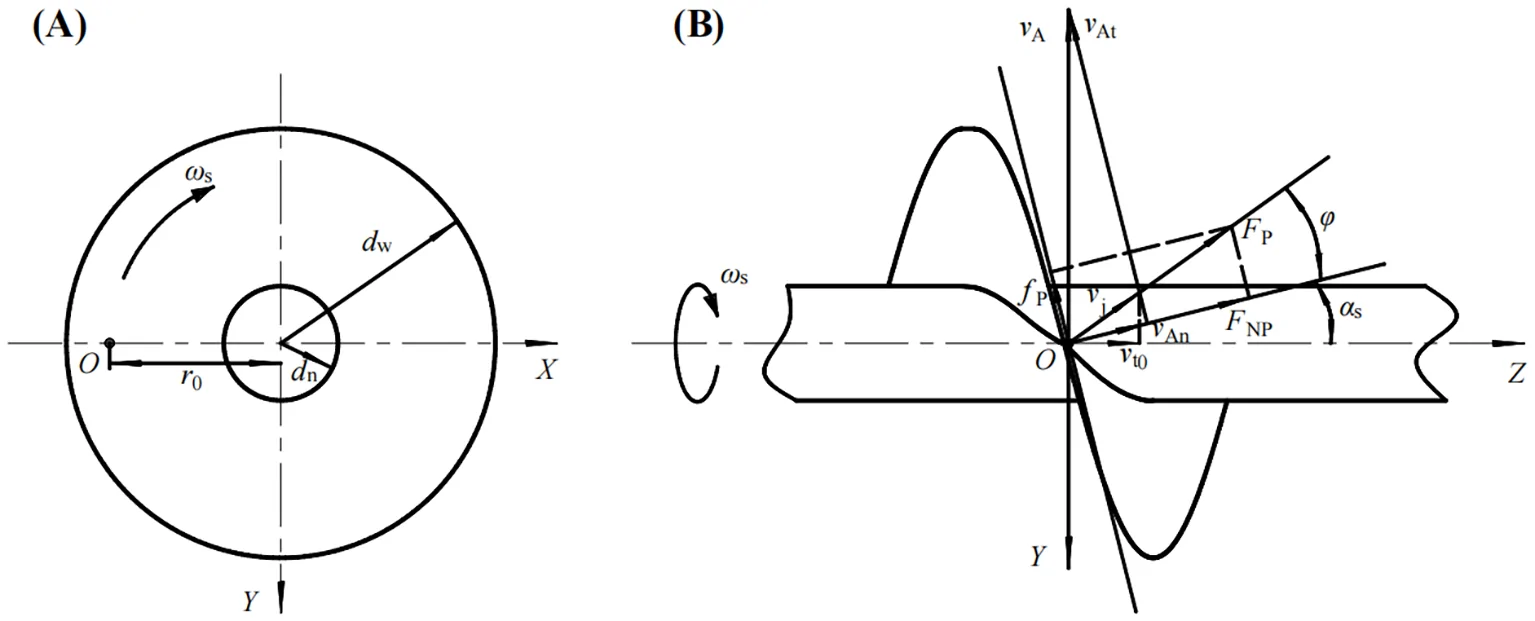
Kinematic analysis of *C. humilis* fruit conveyed by the helical blade. **(A)** Radial cross-section schematic. **(B)** Velocity and force analysis.

To calculate conveying speed, a fruit at radial distance *r*_0_ from the central axis was simplified as particle *O*, while the screw shaft rotated around the *Z* axis at angular velocity *ω*_s_ (**Figure 6A**). The blade helix angle is *α*_s_, the friction angle between fruit and blade is *φ*, *v*_j_ is the absolute velocity, and *v*_a_ is the circumferential velocity of particle *O* (**Figure 6B**).

The circumferential velocity is expressed by Equation 14:

(14) $$v_{\text{A}}=\omega_{\text{s}}r_{0}=\frac{2\pi n_{\text{z}}}{60}\frac{P}{2\pi \tan\alpha_{\text{s}}}=\frac{n_{\text{z}}P}{60\tan\alpha_{\text{s}}}$$

where *n*_z_ is the rotational speed of the screw shaft, r/min; *P* is the lead of the helical blades, m. The normal velocity is expressed by Equation 15:

(15) $$v_{\text{An}}=v_{\text{A}}\sin\alpha_{\text{s}}=\frac{nP}{60}\cos\alpha_{\text{s}}$$

The absolute speed of the fruit is expressed by Equation 16:

(16) $$v_{\text{s}}=\frac{v_{\text{An}}}{\cos\varphi }=\frac{nP\cos\alpha_{\text{s}}}{60\cos\varphi }$$

Therefore, the axial conveying speed of the *C. humilis* fruit is expressed by Equation 17:

(17) $$v_{\text{t0}}=v_{\text{s}}\cos(\alpha_{\text{s}}+\varphi )=\frac{n_{\text{z}}P\cos\alpha_{\text{s}}\cos(\alpha_{\text{s}}+\varphi )}{60\cos\varphi }$$

#### 2.4.3 Structural parameter design of the screw conveyor

**Figure 7:**
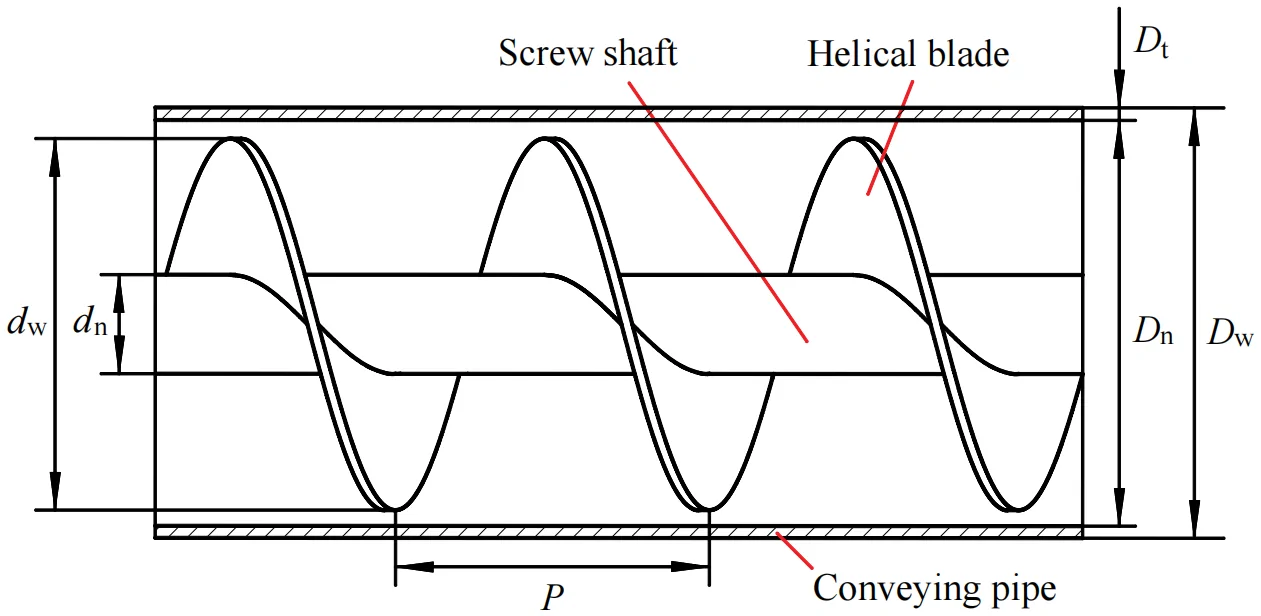
Partial structure of the screw conveyor.

The partial structure of the screw conveyor is shown in **Figure 7**.

The helical-blade pitch P must balance throughput and fruit protection. A larger pitch can increase conveying capacity, but it also raises axial resistance and may intensify fruit compression. A smaller pitch reduces resistance but limits throughput. To allow fruit to pass smoothly, the radial blade width must exceed the maximum fruit diameter *L*_max_, as expressed in Equation 18.

(18) $$2L_{\max}<d_{\text{w}}-d_{\text{n}}$$

where *L*_max_ is the maximum fruit diameter, m; *d*_w_ is the outer diameter of the helical blade, m; *d*_n_ is the inner diameter of the helical blade, m. Based on measurements of mature *C. humilis* fruit, *L*_max_ was 0.0283 m.

Based on the analysis and manufacturing constraints, *d*_w_, *d*_n_ and *P* were set to 0.12 mm, 0.032 m and 0.10 m, respectively. Because fabrication tolerance can change the actual gap, a radial clearance *δ*_i_ was reserved between the blade edge and the conveying-pipe wall. The clearance was required to remain below the values in **Table 1** and above one-half of the nominal clearance.

**Table 1:** Clearance requirements between the helical blade and the inner wall of the conveying pipe.

Item	Parameters							
Outer diameter of the helical blade/mm	100	125	160	200	250	315	400	500
Nominal clearance/mm	7.5	10	10	10	10	10	12.5	12.5

PVC-U pipe was selected for the conveying tube to reduce cost and machine weight while maintaining sufficient strength. Compared to ordinary carbon steel pipes, PVC-U pipes have a lower initial roughness of the inner wall and no risk of rust and deterioration. They can maintain a low-friction conveying interface for a long time, avoiding scratch damage to the skin of the fruit caused by the protrusion of the rust layer and the increase of roughness of the pipe wall, and reducing mechanical damage to the fruit during the transportation process. The selected tube had an outer diameter of 0.14 m and an inner diameter of 0.13 m. Equation 19 gave a 0.005 m radial clearance between the helical blade and the pipe wall, satisfying the design requirement.

(19) $$\delta_{\text{i}}=\frac{D_{\text{n}}-d_{\text{w}}}{2}=0.005\;m$$

### 2.5 Design of the walking chassis

#### 2.5.1 Overall design

**Figure 8:**
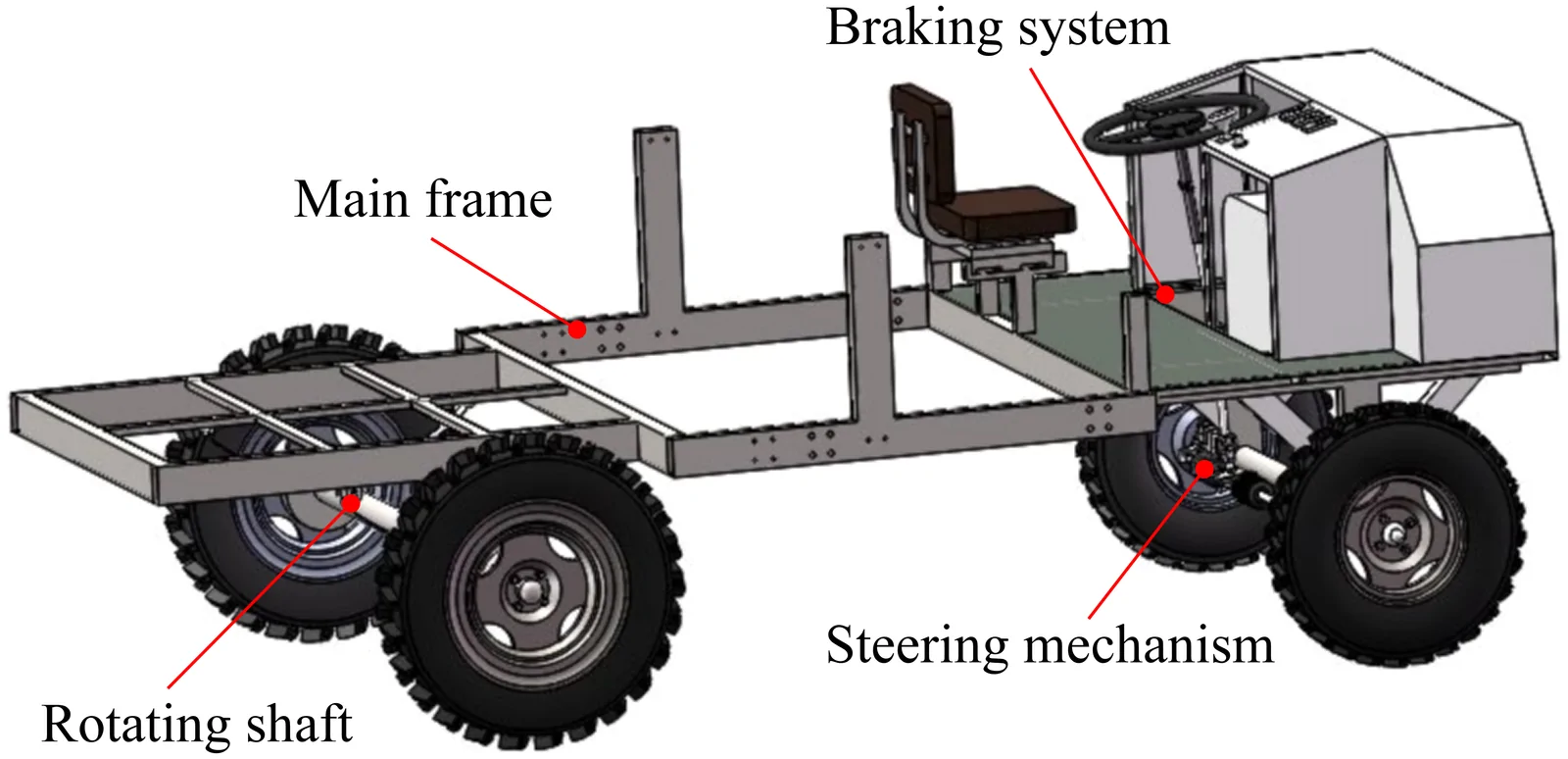
Walking chassis of the *C. humilis* harvester.

The chassis was designed to match the agronomic conditions of *C. humilis* orchards and to provide reliable maneuvering during harvest. A front-wheel steering and rear-wheel drive arrangement was selected for stability and row operation. **Figure 8** shows the main frame, steering mechanism, drive system and braking system.

#### 2.5.2 Frame design and finite element analysis

**Figure 9:**
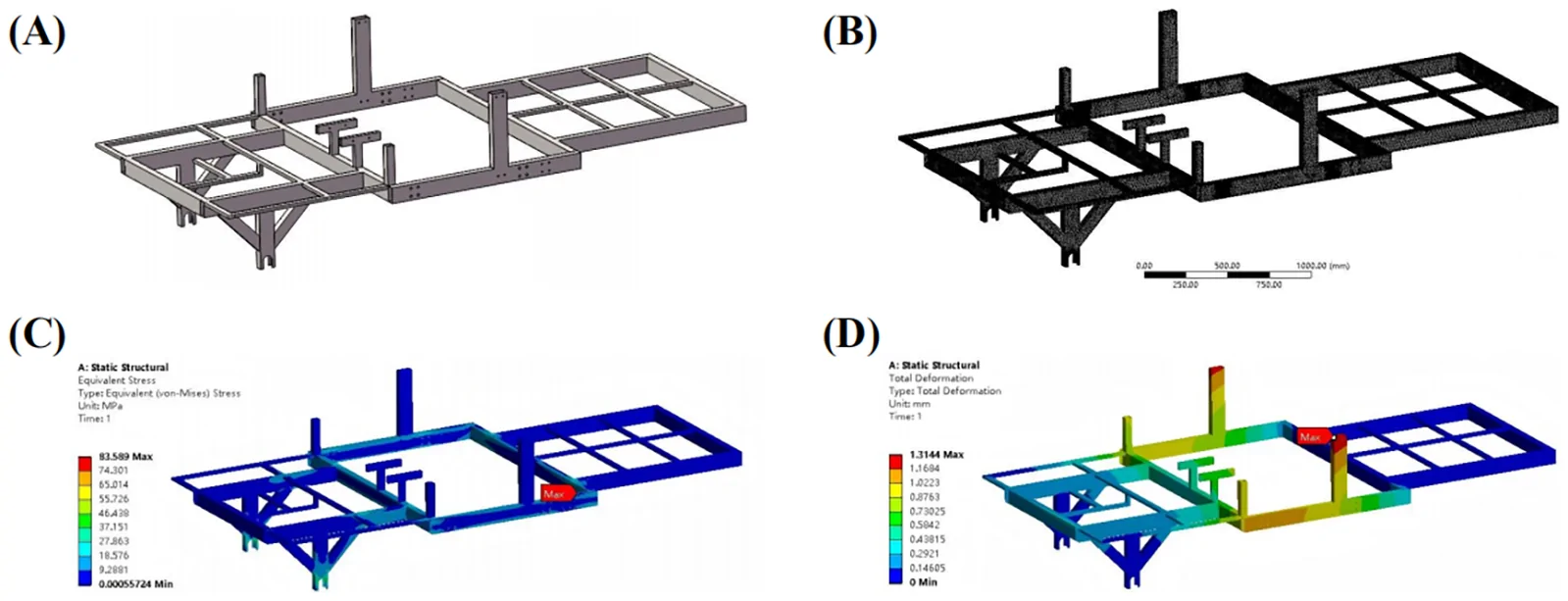
Finite element analysis. **(A)** Three-dimensional model of the main frame. **(B)** Mesh of the main frame. **(C)** Equivalent stress distribution. **(D)** Total deformation distribution.

The frame layout was determined by the installation positions of the functional modules. The front-left region carries the front axle, and the operator platform is placed on the right side. The branch lifter, comb-type stripping device, and screw conveyor are mounted near the middle to balance the machine center of gravity, while the rear section supports the fruit collection bin. To obtain adequate strength with limited mass, the main beams used 100 mm × 50 mm × 4 mm Q235 rectangular steel tubes. The material density, yield strength, elastic modulus, and Poisson ratio were 7.85 g/cm^3^, 235 MPa, 200 GPa and 0.3, respectively. The frame was welded and the three-dimensional model is shown in **Figure 9A**.

ANSYS Workbench 14.0 was used to evaluate frame stiffness and strength under working loads. The three-dimensional frame model was simplified before import, and automatic meshing was applied with local refinement near connection holes. The final mesh contained 501,970 elements and 868,931 nodes (**Figure 9B**). Boundary constraints were imposed at the front-axle positioning points and leaf-spring connections. The applied loads included 750 N at the seat bracket, 5100 N at the mounting holes of the harvesting device, and 5000 N at the fruit collection bin. Static analysis indicated that the largest stress occurred in the rear region of the middle longitudinal beam and reached 83.59 MPa; the maximum deformation was 1.31 mm (**Figures 9C, D**).

The allowable stress of the material was calculated by Equation 20:

(20) $$[\sigma ]=\frac{\sigma_{\text{b}}}{n_{\text{b}}}$$

where [*σ*] is the allowable stress of the material, MPa; *σ*_b_ is the yield strength of the material, designated as 235 MPa; *n*_b_ is the safety factor, which was set to 2. After substituting the material parameters, the allowable stress [*σ*] was 117.5 MPa. The maximum frame stress was 83.59 MPa, which was lower than [*σ*]. The frame therefore met the strength requirement under the defined loading conditions. It should be noted that the above analysis is based on the static rated load condition. In actual field operations, the frame will also be subjected to dynamic loads caused by road surface irregularities, driving vibrations, harvesting operation impacts, and other factors. The maximum static stress of this frame is 83.59 MPa, compared with the allowable stress of 117.5 MPa, resulting in a safety factor of 1.41. The sufficient strength margin can accommodate the increase in dynamic loads under conventional orchard operating conditions. For long-term service scenarios under harsh terrain conditions, further fatigue life analysis and modal analysis will be conducted in future work.

#### 2.5.3 Steering mechanism design and steering-performance analysis

**Figure 10:**
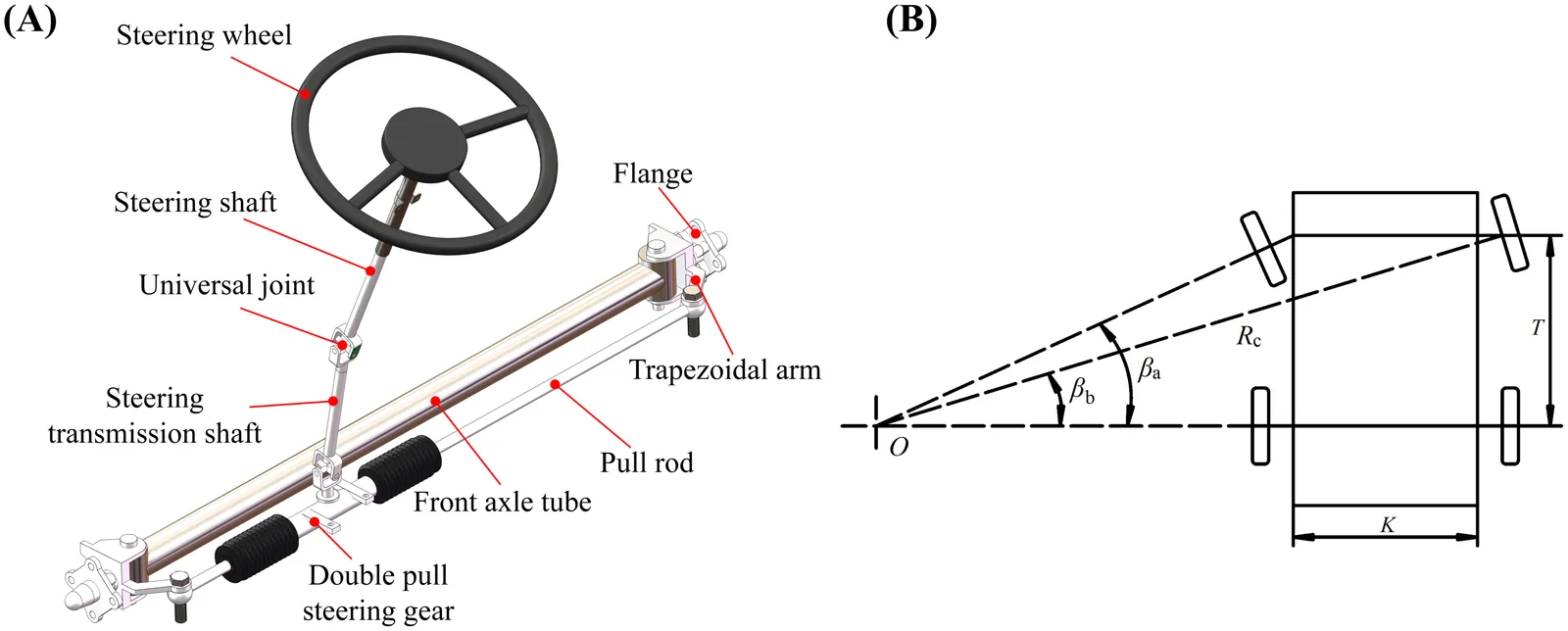
Steering mechanism. **(A)** Structure of the steering mechanism. **(B)** Ackermann steering geometric principle.

The steering mechanism includes the steering wheel, steering shaft, universal joint, steering transmission shaft, double-pull steering gear, pull rods, trapezoidal arms, flanges and front-axle tube (**Figure 10A**). Steering-wheel rotation is converted by the steering gear into pull-rod displacement, which rotates the trapezoidal arms around the front-axle pivots and deflects the front wheels through the flanges. The outer-flange deflection angle ranges from 0° to 45° during turning.

Front-wheel deflection provides steering. To reduce tire slip and wear, the axes of the two front wheels should meet on the rear-axle line during a turn (**Figure 10B**). Based on the steering geometry, the relationship between the front-wheel deflection angles is given in Equation 21:

(21) $$\cot\beta_{\text{b}}-\cot\beta_{\text{a}}=\frac{K}{T}$$

where *β*_b_ is the deflection angle of the outer front wheel, (°); *β*_a_ is the deflection angle of the inner front wheel, (°); *K* is the distance between the front axle pivots, m; *T* is the wheelbase, m.

The minimum turning radius *R*_c_ corresponds to the minimum turning radius of the outer front wheel is given in Equation 22:

(22) $$R_{\text{c}}=\frac{T}{\sin\beta_{\text{b}}}$$

When the distance between the front-axle pivots *K* was 0.619 m and the wheelbase *T* was 2.415 m, the minimum turning radius occurred at the maximum inner front-wheel deflection angle of 45°. Equations 21 and 22 yielded a minimum turning radius of 3.807 m.

### 2.6 Design of the *C. humilis* harvester control system

**Figure 11:**
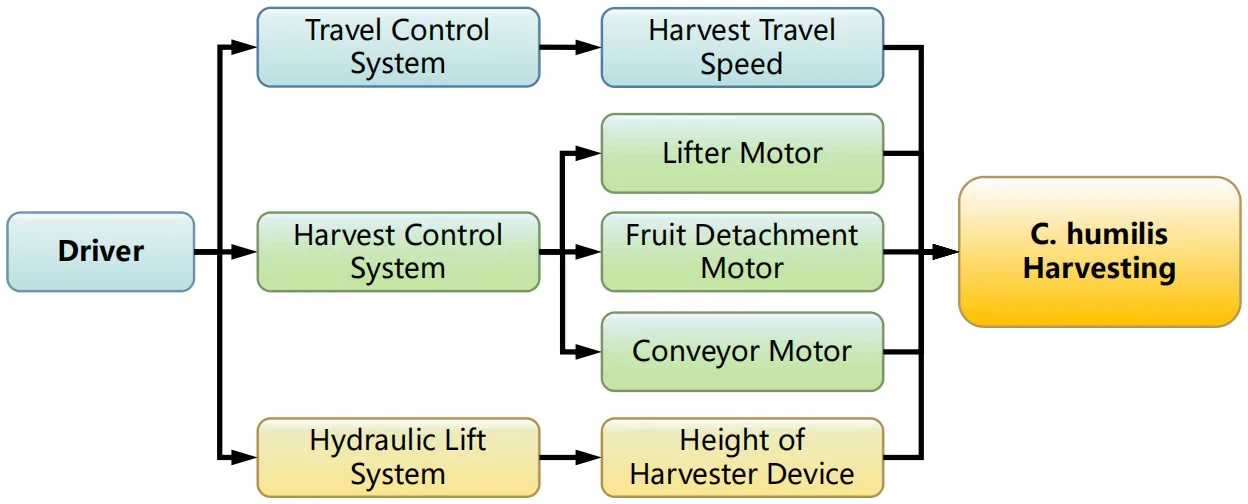
Control system of the *C. humilis* harvester.

The control system links field travel, fruit detachment, fruit conveying and working-height adjustment. It includes travel control, harvesting control, and hydraulic lifting subsystems. The driver coordinates machine travel and harvesting through the central control system shown in **Figure 11**.

The travel control subsystem contains control switches, an electronic accelerator pedal, a brushless DC motor controller, a lead-acid battery pack and a drive motor. After the main power is switched on, the driver selects the travel direction and speed range. Pedal position changes the Hall-sensor voltage signal, and the controller uses this signal to regulate the drive motor. The harvesting control subsystem contains an AC generator, frequency converters, and motors for branch lifting, fruit stripping and conveying. The three frequency converters independently regulate module speed over a 0–1500 r/min range. Independent speed regulation provides the hardware basis for coordinating branch posture, detachment intensity and fruit transport. The frequency converters exhibit a dynamic response time of less than 200 ms, ensuring rapid speed adjustments and maintaining steady-state synchronization between the branch-lifting and fruit-stripping modules. Furthermore, the system is powered by a 48V, 120Ah lead-acid battery pack. Under full-load field operations encompassing simultaneous driving, lifting, stripping, and conveying, the estimated continuous endurance reaches approximately 3 hours.

**Figure 12:**
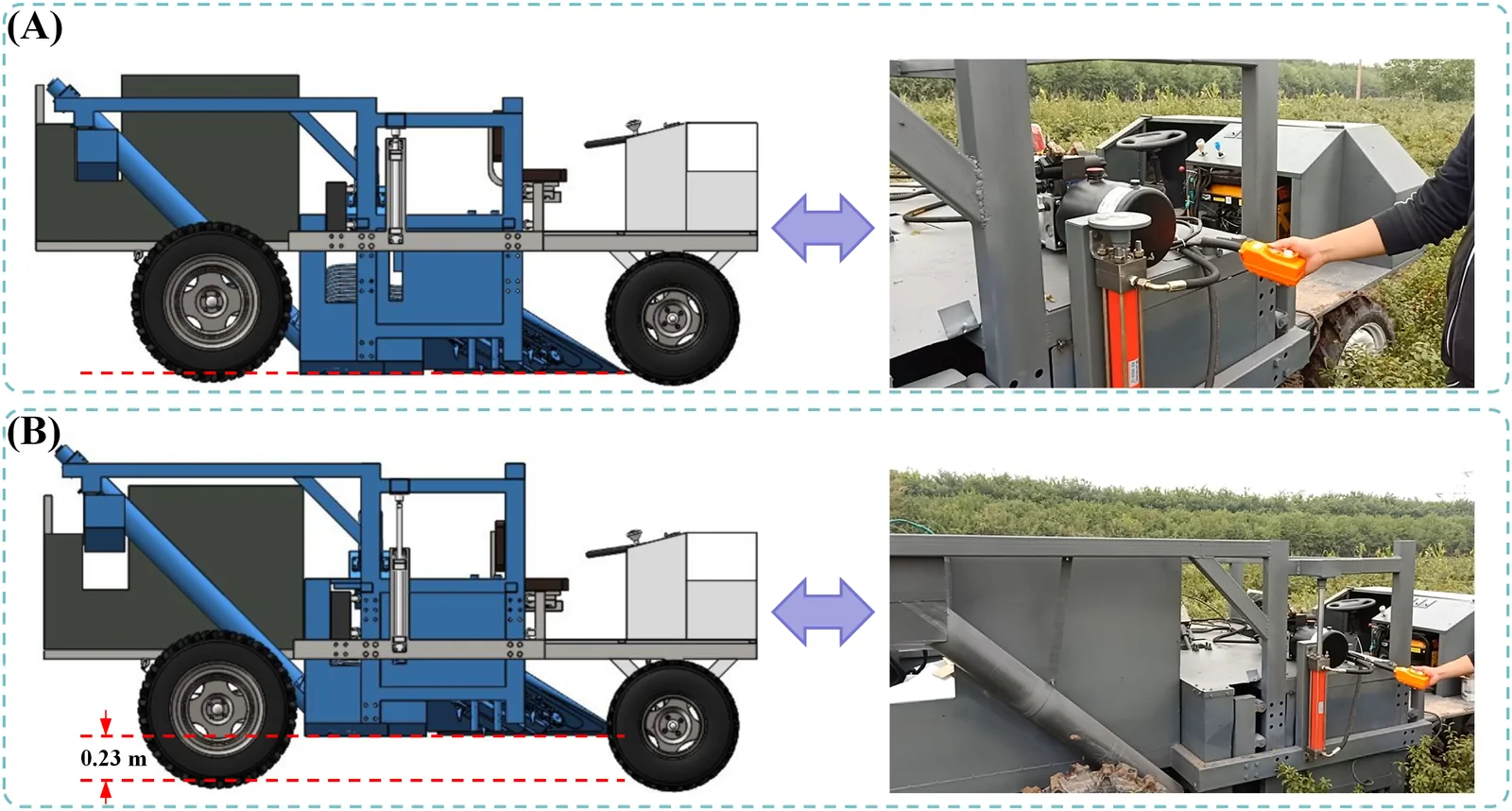
Hydraulic lifting positions. **(A)** Lowest position. **(B)** Highest position.

During harvesting, the integrated working assembly must reach the lower portions of lodged branches. A hydraulic lifting system was therefore added to adjust the vertical position of the branch lifter, stripping device and screw conveyor. These components are mounted on a rectangular-tube frame: side support plates carry the load, and eight guide wheels on the main frame stabilize vertical motion. The designed mass of the assembly ${\text{m}}_{\text{z}}$ was 509.4 kg, and the lifting stroke was 0-0.23 m. **Figure 12A** is the lowest position, and **Figure 12B** is the highest position.

Considering the load and required stroke, a single-piston-rod double-acting hydraulic cylinder (HOB40 × 300) was selected. The bore diameter, piston-rod diameter, and maximum stroke were 40 mm, 25 mm and 300 mm, respectively. The working pressure was calculated with Equation 23.

(23) $$p=\frac{F_{\text{z}}}{A}=\frac{2m_{\text{z}}g}{\pi D^{2}}$$

where *p* is the oil pressure in the rodless chamber, Pa; *F*_z_ is the total gravitational force of the integrated assembly, N; *A* is the cylinder-bore cross-sectional area, m^2^; *m*_z_ is the designed assembly mass, kg; *g* is gravitational acceleration, 9.8 m/s^2^; *D* is the cylinder inner diameter, m. Substitution of the known parameters gave a working pressure of 1.986 MPa.

To ensure smooth ascent of the harvesting assembly, the piston-rod extension speed must be controlled. The maximum ascent velocity was determined as follows:

(24) $$v_{\text{z}}=\frac{Q_{\text{b}}}{2A}=\frac{2q_{\text{p}}n_{\text{z}}}{\pi D^{2}}$$

where *v*_z_ is the maximum upward velocity, m/s; *Q*_b_ is the output flow rate of the hydraulic pump, m^3^/s; *q*_p_ is the pump displacement, m^3^/s; *n*_z_ is the rated rotational speed of the hydraulic pump motor, r/min. Substitution into Equation 24 gave a maximum piston-rod extension velocity of 0.01 m/s.

**Figure 13:**
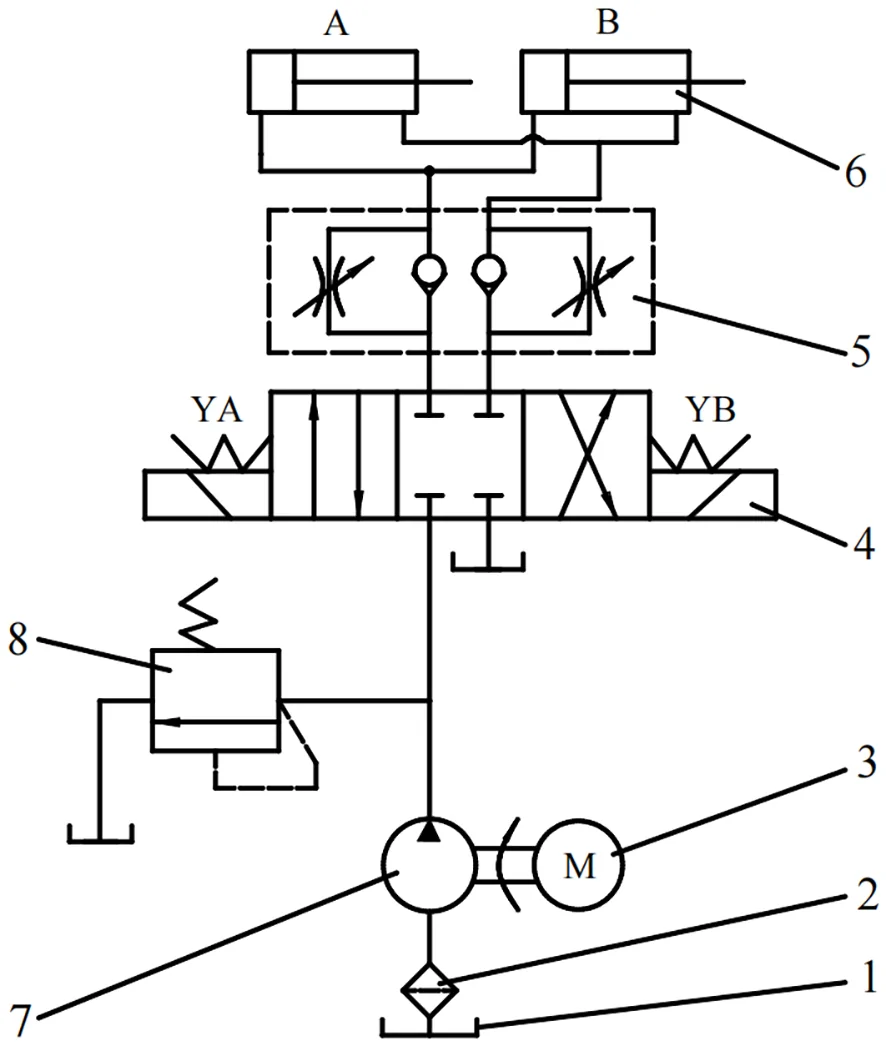
Hydraulic circuit for the lifting and lowering system. 1. hydraulic reservoir 2. filter 3. electric motor 4. three-position four-way solenoid directional valve 5. adjustable one-way throttle valve 6. single-piston-rod double-acting hydraulic cylinder 7. unidirectional fixed-displacement hydraulic pump 8. relief valve.

**Figure 13** shows the hydraulic circuit used for lifting and lowering. Energizing solenoid YA of the three-position four-way directional valve extends the piston rod and raises the harvesting assembly; energizing solenoid YB lowers the assembly.

The performance of the hydraulic lifting mechanism was verified through bench tests. Under full-load conditions, the actual effective lifting stroke was 22.7 cm, with a relative error of 1.3% compared to the designed stroke. The average lifting speed was 0.98 cm per second, and the repeat positioning accuracy was ±0.47 cm. After 30 consecutive lifting cycle tests, no jamming or hydraulic leakage occurred, indicating good operational stability and meeting the height adjustment requirements for field harvesting.

### 2.7 Field test method for harvesting performance

**Figure 14:**
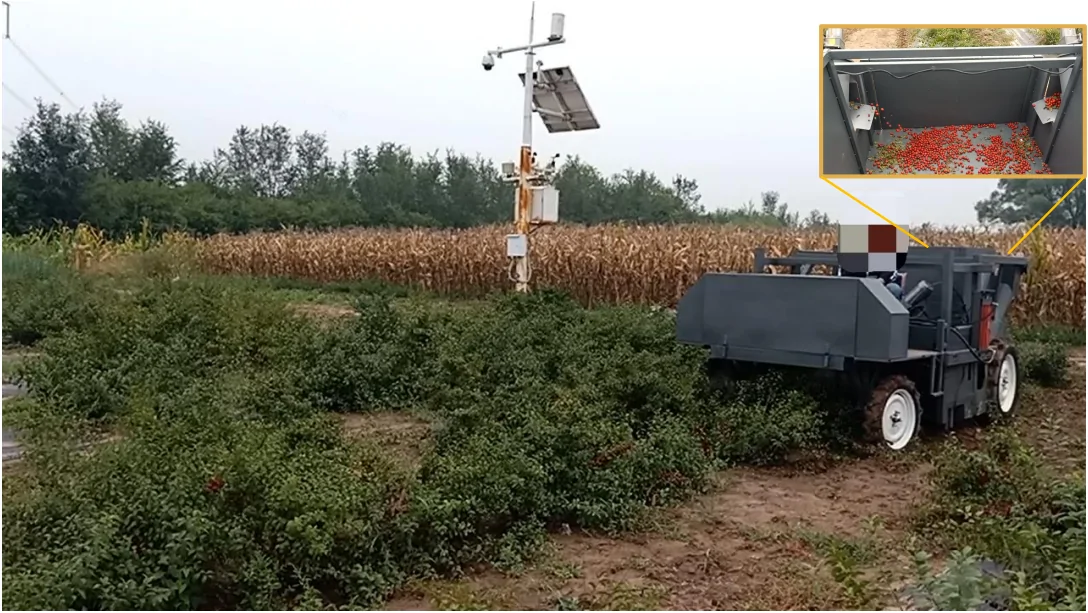
Field test sites.

Field experiments were conducted at the Juxin Modern Agricultural Base in Taigu County using the ‘Nongda No. 6’ cultivar. The plant spacing of the experimental plot is 0.5 m, the narrow row spacing is 0.5 m, the wide row spacing is 1.2 m, the branch length is 0.6~0.8 m, and the angle between the fruiting branches and the ground after natural fall is 30°~65°. The test equipment included the self-propelled electric harvester prototype and an electronic balance with 0.1 g precision. The field test site is shown in **Figure 14**.

The tested factors were harvester travel speed (*x*_1_), branch-lifter motor speed (*x*_2_), fruit-stripping motor speed (*x*_3_) and conveying motor speed (*x*_4_). The parameter ranges for the field experiments were determined based on preliminary single-factor bench tests. These initial tests indicated that operating outside these selected ranges resulted in extreme outcomes: excessively low speeds caused severe branch jamming and minimal fruit detachment, whereas excessively high speeds led to unacceptable mechanical bruising and severe fruit scattering. The level ranges of the four factors were determined based on preliminary field tests and previous research on *C. humilis* harvesting mechanisms (He et al., 2018; Kang et al., 2024), as well as reference to operating parameters of similar shrub fruit harvesters (Chen et al., 2024a; Du et al., 2021). Travel speed was set at 0.2-0.8 m/s to balance operation efficiency and harvesting quality; branch-lifter speed was 80–140 r/min to ensure effective branch lifting while reducing fruit impact; stripping speed was 15–35 r/min according to single-branch combing test results; conveying speed was 90–150 r/min to avoid blockage and excessive conveying damage. A four-factor, three-level Box-Behnken design was used to evaluate their combined effects on harvesting performance. The coded levels are shown in **Table 2**.

**Table 2:** Encoding values of the factor levels.

Coded level	*x*_1_/(m/s)	*x*_2_/(r/min)	*x*_3_/(r/min)	*x*_4_/(r/min)
-1	0.2	80	15	90
0	0.5	110	25	120
1	0.8	140	35	150

Harvesting rate (*Y*_1_) and fruit damage rate (*Y*_2_) were used as performance indicators because they represent the two main requirements of low-damage field harvesting. In this study, damaged fruit is defined as fruit with visible epidermal damage, indented indentations in the flesh, or tears at the stem end. Damage assessment is conducted by three trained experimental personnel who independently visually verify the results and obtain a consensus. The indicators were calculated with Equations 25 and 26. Each treatment was performed three times, and the mean value was used for model fitting and analysis.

(25) $$Y_{1}=\frac{M_{1}}{M}\times 100\%$$

(26) $$Y_{2}=\frac{M_{2}}{M_{1}}\times 100\%$$

where *M* is the total fruit mass, kg; *M*_1_ is the mass of fruit collected in the collection bin, kg; *M*_2_ is the mass of damaged fruit among the collected fruit, kg.

## 3 Results

### 3.1 Regression model establishment and analysis of variance

**Table 3** summarizes the experimental design and measured responses. Design-Expert 8.0.5 was used to fit quadratic response surface models for harvesting rate (*Y*_1_) and damage rate (*Y*_2_) from the coded factors. The resulting models are shown in Equations 27 and 28, and analysis of variance was used to test model significance and factor effects (**Tables 4**, **5**).

**Table 3:** Test program and results.

No.	*x*_1_	*x*_2_	*x*_3_	*x*_4_	*Y*_1_/%	*Y*_2_/%	No.	*x*_1_	*x*_2_	*x*_3_	*x*_4_	*Y*_1_/%	*Y*_2_/%
1	1	-1	0	0	70.63	5.12	16	1	1	0	0	73.95	5.58
2	0	-1	-1	0	68.06	3.68	17	-1	0	-1	0	76.5	2.25
3	-1	-1	0	0	77.15	3.17	18	0	0	0	0	78.83	4.13
4	0	1	-1	0	71.34	4.54	19	1	0	-1	0	70.01	2.39
5	0	-1	0	1	73.55	4.62	20	0	1	0	1	75.18	7.85
6	0	1	0	-1	75.75	5.51	21	0	0	1	1	74.35	9.18
7	0	0	0	0	79.93	4.31	22	-1	1	0	0	78.12	5.63
8	-1	0	0	-1	80.25	4.32	23	0	0	0	0	80.03	4.54
9	0	0	-1	-1	73.82	2.33	24	1	0	1	0	73.15	6.33
10	0	0	-1	1	73.6	3.81	25	-1	0	1	0	76.53	5.38
11	1	0	0	1	76.25	4.65	26	0	0	0	0	80.13	4.8
12	0	0	0	0	79.73	3.82	27	0	-1	0	-1	75.13	6.15
13	1	0	0	-1	76.83	2.28	28	0	0	1	-1	74.95	4.25
14	-1	0	0	1	82.51	4.09	29	0	1	1	0	72.07	10.27
15	0	-1	1	0	71.45	5.3							

**Table 4:** Analysis of variance for the *C. humilis* harvesting rate regression model.

Source of variance	Sum of squares	Degrees of freedom	Mean square	*F* value	*P* value
Model	346.07	14	24.72	70.02	< 0.0001**
${\text{x}}_{1}$	76.20	1	76.20	215.85	< 0.0001**
${\text{x}}_{2}$	9.08	1	9.08	25.73	0.0002**
${\text{x}}_{3}$	7.01	1	7.01	19.85	0.0005**
${\text{x}}_{4}$	0.14	1	0.14	0.39	0.5409
${\text{x}}_{1}{\text{x}}_{2}$	1.38	1	1.38	3.91	0.0680
${\text{x}}_{1}{\text{x}}_{3}$	2.42	1	2.42	6.85	0.0203*
${\text{x}}_{1}{\text{x}}_{4}$	2.02	1	2.02	5.71	0.0315*
${\text{x}}_{2}{\text{x}}_{3}$	1.77	1	1.77	5.01	0.0420*
${\text{x}}_{2}{\text{x}}_{4}$	0.26	1	0.26	0.72	0.4097
${\text{x}}_{3}{\text{x}}_{4}$	0.04	1	0.04	0.10	0.7539
${\text{x}}_{1}^{2}$	1.69	1	1.69	4.79	0.0461
${\text{x}}_{2}^{2}$	114.31	1	114.31	323.77	< 0.0001**
${\text{x}}_{3}^{2}$	163.25	1	163.25	462.39	< 0.0001**
${\text{x}}_{4}^{2}$	1.46	1	1.46	4.13	0.0615
Residual	4.94	14	0.35		
Lack of fit	3.84	10	0.38	1.40	0.3999
Pure error	1.10	4	0.28		
Cor total	351.01	28			

** indicates extremely significant (*P* < 0.01); *Significant (*P* < 0.05).

**Table 5:** Analysis of variance for the *C. humilis* damage rate regression model.

Source of variance	Sum of squares	Degrees of freedom	Mean square	*F* value	*P* value
Model	89.39	14	6.38	10.95	< 0.0001**
${\text{x}}_{1}$	0.19	1	0.19	0.33	0.5771
${\text{x}}_{2}$	10.72	1	10.72	18.38	0.0008**
${\text{x}}_{3}$	39.28	1	39.28	67.37	< 0.0001**
${\text{x}}_{4}$	7.30	1	7.30	12.52	0.0033**
${\text{x}}_{1}{\text{x}}_{2}$	1.00	1	1.00	1.72	0.2114
${\text{x}}_{1}{\text{x}}_{3}$	0.16	1	0.16	0.28	0.6041
${\text{x}}_{1}{\text{x}}_{4}$	1.69	1	1.69	2.90	0.1107
${\text{x}}_{2}{\text{x}}_{3}$	4.22	1	4.22	7.24	0.0175*
${\text{x}}_{2}{\text{x}}_{4}$	3.74	1	3.74	6.42	0.0238*
${\text{x}}_{3}{\text{x}}_{4}$	2.98	1	2.98	5.10	0.0403*
${\text{x}}_{1}^{2}$	3.24	1	3.24	5.55	0.0336*
${\text{x}}_{2}^{2}$	11.34	1	11.34	19.46	0.0006**
${\text{x}}_{3}^{2}$	0.83	1	0.83	1.43	0.2513
${\text{x}}_{4}^{2}$	0.49	1	0.49	0.84	0.3745
Residual	8.16	14	0.58		
Lack of fit	7.60	10	0.76	5.38	0.0596
Pure error	0.57	4	0.14		
Cor total	97.55	28			

** indicates extremely significant (*P* < 0.01); *Significant (*P* < 0.05).

(27) $$Y_{1}=79.73-2.52x_{1}+0.87x_{2}+0.76x_{3}+0.78x_{1}x_{3}-0.71x_{1}x_{4}-0.67x_{2}x_{3}-4.20{x^{2}}_{2}-5.02{x^{2}}_{3}$$

(28) $$Y_{2}=4.32+0.94x_{2}+1.81x_{3}+0.78x_{4}+1.03x_{2}x_{3}+0.97x_{2}x_{4}+0.86x_{3}x_{4}-0.71{x^{2}}_{1}+1.32{x^{2}}_{2}$$

Analysis of variance showed that both regression models were highly significant (*P* < 0.01), whereas the lack-of-fit terms were not significant (*P* > 0.05). The coefficients of determination were 0.9859 for the harvesting-rate model and 0.9163 for the damage-rate model. Thus, the fitted models explained most variation in the two performance indicators within the tested parameter range.

For harvesting rate, the first-order terms ${\text{x}}_{1}$, ${\text{x}}_{2}$ and ${\text{x}}_{3}$ were significant, whereas ${\text{x}}_{4}$ was not significant. The quadratic terms ${\text{x}}_{2}^{2}$ and ${\text{x}}_{3}^{2}$ were extremely significant, and ${\text{x}}_{1}^{2}$ was significant. The interaction terms ${\text{x}}_{1}{\text{x}}_{3}$, ${\text{x}}_{1}{\text{x}}_{4}$ and ${\text{x}}_{2}{\text{x}}_{3}$ were significant. Based on the *F* values of the first-order terms, the effect order was ${\text{x}}_{1}$ > ${\text{x}}_{2}$ > ${\text{x}}_{3}$ > ${\text{x}}_{4}$.

For damage rate, the first-order terms ${\text{x}}_{2}$, ${\text{x}}_{3}$ and ${\text{x}}_{4}$ were significant, whereas ${\text{x}}_{1}$ was not significant. The quadratic term ${\text{x}}_{2}^{2}$ was extremely significant, and ${\text{x}}_{1}^{2}$ was significant. The interaction terms ${\text{x}}_{2}{\text{x}}_{3}$, ${\text{x}}_{2}{\text{x}}_{4}$ and ${\text{x}}_{3}{\text{x}}_{4}$ were significant. Based on the *F* values of the first-order terms, the effect order was ${\text{x}}_{3}$ > ${\text{x}}_{2}$ > ${\text{x}}_{4}$ > ${\text{x}}_{1}$.

### 3.2 Effects of interaction factors on harvesting performance

**Figure 15:**
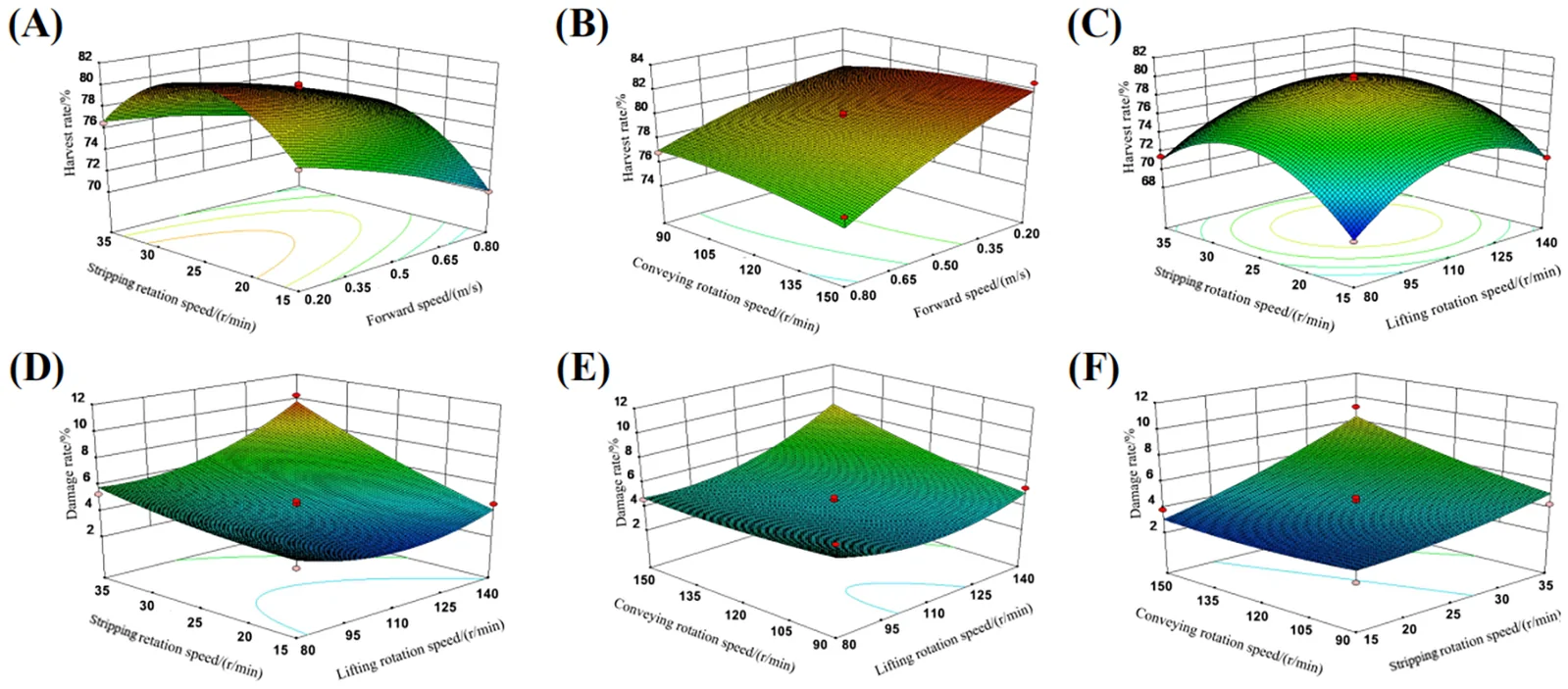
Response surface plots of *C. humilis* harvesting performance. **(A)** Impact of ${\text{x}}_{1}{\text{x}}_{3}$ on the harvesting rate. **(B)** Impact of ${\text{x}}_{1}{\text{x}}_{4}$ on the harvesting rate. **(C)** Impact of ${\text{x}}_{2}{\text{x}}_{3}$ on the harvesting rate. **(D)** Impact of ${\text{x}}_{2}{\text{x}}_{3}$ on the damage rate. **(E)** Impact of ${\text{x}}_{2}{\text{x}}_{4}$ on the damage rate. **(F)** Impact of ${\text{x}}_{3}{\text{x}}_{4}$ on the damage rate.

Quadratic response surface plots were generated to visualize the effects of the operating factors on harvesting rate and damage rate (**Figure 15**).

The response surfaces showed how the operating parameters controlled fruit detachment and damage. Harvesting rate increased and then declined as stripping rotation speed increased from 15 r/min to 35 r/min (**Figure 15A**). Low stripping speed produced too few effective comb contacts, whereas excessive speed increased impact velocity and fruit scattering. Conveying speed had little direct influence on harvesting rate after travel speed was considered because conveying occurred after fruit separation (**Figure 15B**). A similar increase-then-decrease response occurred when branch-lifter and stripping rotation speeds increased together, indicating that branch guidance and stripping must be synchronized (**Figure 15C**). Damage rate increased when branch-lifter, stripping, and conveying speeds increased in interacting combinations, consistent with the significant ${\text{x}}_{2}{\text{x}}_{3}$, ${\text{x}}_{2}{\text{x}}_{4}$ and ${\text{x}}_{3}{\text{x}}_{4}$ terms in the ANOVA (**Figures 15D–F**).

### 3.3 Parameter optimization and validation experiments

Numerical optimization in Design-Expert 8.0.5 searched for a parameter combination that increased harvesting rate while reducing fruit damage. The optimized settings were 0.2 m/s travel speed, 104.6 r/min branch-lifter speed, 22 r/min stripping rotation speed, and 137.4 r/min conveying motor speed. The corresponding predicted harvesting rate and damage rate were 81.26% and 2.80%, respectively. Five independent field validation tests were then conducted under these settings, and the results are listed in **Table 6**.

**Table 6:** Verification test results.

No.	Tests results		Relative error of harvesting rate/%	Relative error of damage rate/%
	*Y*_1_/%	*Y*_2_/%		
Predictive value	81.26	2.80	0.00	0.00
1	79.31	2.70	2.46	3.70
2	83.05	2.93	2.16	4.44
3	84.16	2.72	3.45	2.94
4	80.12	2.89	1.42	3.11
5	83.96	2.72	3.22	2.94
Mean ± SD	± 2.25	± 0.11	2.54	3.43
95% CI	[79.33%, 84.91%]	[2.65%, 2.93%]	–	–

The validation tests produced a mean harvesting rate of 82.12% and a mean damage rate of 2.79%. The mean relative errors were 2.54% for harvesting rate and 3.43% for damage rate, while the maximum relative errors were 3.45% and 4.44%, respectively. Based on the five independent validation tests, the 95% confidence interval (CI) for the harvesting rate was [79.33%, 84.91%], and for the damage rate was [2.65%, 2.93%]. These results indicate that the response surface models predicted low-damage harvesting performance with acceptable accuracy within the tested range. In addition to the harvesting rate and damage rate, the field operation performance of the prototype was also further evaluated. Under the optimized parameter conditions, the actual field operation capacity (including inter-row transfer) was approximately 0.08 ha/h, and the full battery endurance time was 2.5 hours. The operation efficiency of this prototype is equivalent to that of about 6 manual laborers, and it has a significant labor-saving advantage in short harvest periods. Economically, although the prototype’s estimated manufacturing cost is approximately 25000 RMB, its field capacity is equivalent to 6 manual laborers. Given local manual harvesting costs of 300 RMB/d, a mid-scale orchard can recover the investment within one harvest seasons.

## 4 Discussion

### 4.1 System performance and comparative analysis

This study reframes *C. humilis* harvesting as a coordinated plant-production problem rather than a single detachment operation. The prototype integrates branch lifting, comb-type stripping, screw conveying, self-propelled travel, and electrohydraulic height adjustment in one field-operable electric system. Compared with earlier *C. humilis* studies focused on fruit mechanics, impact behavior, or single-branch combing, the present work connects the main harvesting stages and evaluates their combined field performance.

The results show that low-damage harvesting depends on balancing effective mechanical contact with excessive impact. Travel speed was the dominant factor for harvesting rate because it determines how long branches remain in the stripping zone. Stripping rotation speed was the dominant factor for damage rate because it directly changes the collision velocity between comb teeth and fruit. These findings support coordinated branch lifting and controlled comb-type stripping, rather than simply increasing motor speeds to improve detachment. Detailed classification and quantitative analysis of fruit damage types will be carried out in future work, combined with high-speed photography and image detection technology, to further reveal the damage mechanism of each working link and support more precise low-damage optimization.

The screw conveyor mainly affected fruit damage rather than harvesting rate, which is important for system optimization. Fruit detachment and fruit transport should be tuned separately because they act on different stages of the harvest process. Conveying speed must be high enough to prevent fruit accumulation in the receiving bin, but low enough to limit compression and secondary impact. The response surface results therefore provide operating ranges for balancing field capacity and fruit quality. The harvest rate first increases and then decreases with the combing speed, which is consistent with the parameter change trend of tea oil twisting and combing harvesting (Wu et al., 2021). Too low a speed of the actuator will reduce the number of effective actions, while too high a speed will exacerbate the ejection of the fruit; and the characteristic of the damage rate increasing monotonically with the speed is also consistent with the damage pattern of mechanized blueberry harvesting (Li et al., 2024). The main loss sources include un-lifted low branches, inner canopy fruits untouched by comb teeth, rebound scattering and small fruit leakage. Correspondingly, future improvements will focus on optimizing lifting finger geometry to enhance the lifting effect of ground-hugging branches, increasing comb coverage for inner canopy fruits, adding flexible bin baffles to reduce rebound loss, and matching comb spacing to the fruit diameter range of target cultivars.

The electric-control architecture also provides a practical basis for future sensor-guided harvesting systems. Although the present prototype did not include perception, autonomous navigation, or closed-loop speed control, independent motor regulation and electrohydraulic height adjustment create a modular platform for later upgrades. Future work could add sensing, adaptive parameter control, and route automation to improve operation under variable canopy and field conditions. This positioning aligns the system with sustainable mechanization while keeping the claims within the evidence of the field tests.

As shown in **Table 7**, compared with previous bench-scale *C. humilis* harvesting studies (Liu et al., 2021), this work realizes the integrated self-propelled field operation of branch lifting, comb stripping and conveying for the first time, with better damage performance than single-mechanism test benches. Compared with similar shrub-fruit harvesters such as *Lycium barbarum* L (Chen et al., 2024a). and Oil-tea camellia fruit equipment (Du et al., 2021), the system adds a targeted branch-lifting module to adapt to severely lodged branches, and its damage rate reaches the advanced level of comb-type harvesting devices. Specifically, the proposed system achieved the lowest damage rate (2.79%) among the compared field platforms, notably outperforming both the walk-behind torsional vibration harvester (3.70%) and the vision-guided robotic end-effector (13.30%). Although its harvesting rate (82.12%) is slightly lower than those of bench-scale or walk-behind mechanisms (>93%), it effectively balances the trade-off between continuous self-propelled throughput and strict fruit protection. In contrast to vision-guided robotic harvesting schemes (Paul et al., 2026), the proposed mechanized system has higher throughput and lower cost, which is more compatible with the large-scale and short-harvest-window production characteristics of commercial *C. humilis* orchards.

**Table 7:** Comparison of typical fruit harvesting systems with the proposed harvester.

Harvesting system	Target crop	Automation level	Harvesting rate/%	Damage rate/%
Comb-type harvesting test bench (Liu et al., 2021)	*C. humilis*	Bench-scale, manual feeding	93.82	–
Torsional vibration harvester (Chen et al., 2024a)	*Lycium barbarum* L.	Walk-behind	95.67	3.70
Variable-spacing comb-brush harvester (Du et al., 2021)	Oil-tea camellia fruit	Vehicle-mounted	80.00	–
Vision-guided robotic end-effector (Paul et al., 2026)	Greenhouse capsicum	Robotic autonomous operation	93.30	13.30
Proposed system	*C. humilis*	Self-propelled electric	82.12	2.79

### 4.2 Limitations and future perspectives

The field tests demonstrated the system’s viability, but its performance remains sensitive to agronomic and environmental variability. Unripe fruit resists comb-type stripping, whereas overripe fruit is highly susceptible to mechanical injury. Severe lodging and heavy branch entanglement frequently fall outside the capture range of the lifting fingers, constituting the primary cause of unharvested losses. Soil hardness affects chassis kinematics: soft or uneven terrain increases drive-wheel slip, disrupting the critical speed synchronization required for low-damage harvesting. The modular electric control architecture provides a good foundation for intelligent upgrades. Recent developments in vision-guided 6-DOF robotic arms (Paul and Machavaram, 2025a), ROS-based kinematic simulations (Paul and Machavaram, 2025b), and DEM evaluations of storage dynamics (Paul and Machavaram, 2025c) offer valuable structural and algorithmic frameworks for these future upgrades. Reserved sensor installation positions and CAN bus communication interfaces support the subsequent integration of machine vision for canopy perception and fruit identification, autonomous navigation for row operation, and intelligent decision-making algorithms for adaptive parameter adjustment, so as to further improve harvesting efficiency and adaptability under variable field conditions. Further work will address these constraints by refining lifting-finger geometry and integrating machine vision to enable real-time adaptive parameter control. The current field validation was conducted only on the ‘Nongda No. 6’ cultivar at a single site, so the generalizability of the results has certain boundaries. Differences in cultivar, canopy structure, maturity stage and orchard terrain will affect harvesting performance to varying degrees. For cultivars with different fruit sizes or peel strength, comb spacing and stripping speed need to be calibrated; for severely lodged or dense canopies, operation parameters should be adjusted accordingly. Multi-site and multi-cultivar tests will be carried out in future work to expand the application scope of the system.

## 5 Conclusions

This study developed a low-damage self-propelled electric harvesting system for lodging-prone *C. humilis* and evaluated its coordinated field performance. The system integrates branch lifting, comb-type stripping, screw conveying, self-propelled travel and electrohydraulic height adjustment. The design addresses the crop-specific requirement of raising lodged branches before detachment and conveying fruit with limited secondary impact.

Response surface analysis identified travel speed as the main factor affecting harvesting rate and stripping rotation speed as the main factor affecting damage rate. The optimized settings were 0.2 m/s travel speed, 104.6 r/min branch-lifter speed, 22 r/min stripping rotation speed and 137.4 r/min conveying motor speed. Five validation tests under these settings produced a mean harvesting rate of 82.12% and a mean damage rate of 2.79%, close to the predicted values of 81.26% and 2.80%.

These findings provide a prototype and parameter basis for sustainable mechanized harvesting of *C. humilis* under the tested cultivar and site conditions. Broader validation across cultivars, canopy structures, maturity stages, sites and operating durations is still needed before large-scale application.

## Data Availability

The original contributions presented in the study are included in the article/supplementary material, further inquiries can be directed to the corresponding author/s.
